# Optimization of Quartz Sand-Enhanced Coagulation for Sewage Treatment by Response Surface Methodology

**DOI:** 10.3390/ma17143482

**Published:** 2024-07-14

**Authors:** Zhengan Zhang, Yepu Li, Yongzhi Liu, Yuying Li, Zonghua Wang, Dayang Wang, Lu Yan, Jiayin Zhao, Bailian Larry Li

**Affiliations:** 1 Henan International Joint Laboratory of Watershed Ecological Security in the Water Source Area of the Middle Route of South-to-North Water Diversion Project, College of Water Resource and Modern Agriculture, Nanyang Normal University, Nanyang 473061, China; zhangzhengan0397@163.com (Z.Z.); ypli2015@lzu.edu.cn (Y.L.); lyying200508@163.com (Y.L.); wangzonghua3511@163.com (Z.W.); wangdy58@mail2.sysu.edu.cn (D.W.); yanlu0507@126.com (L.Y.); bai-lian.li@ucr.edu (B.L.L.); 2 School of Environmental Engineering, Henan University of Technology, Zhengzhou 450001, China; 3 College of Water Resource and Modern Agriculture, Nanyang Normal University, Nanyang 473061, China; zhaojiayin040508@163.com; 4 Department of Botany and Plant Sciences, University of California, Riverside, CA 92521, USA

**Keywords:** quartz sand, turbidity, coagulation, response surface methodology, polyaluminum chloride

## Abstract

The quartz sand-enhanced coagulation (QSEC) is an improved coagulation method for treating water, which uses quartz sand as a heavy medium to accelerate the sedimentation rate of flocs and reduce the sedimentation time. The factors that influence the QSEC effect and can be controlled manually include the quartz sand dosage, coagulant dosage, sewage pH, stirring time, settling time, etc., and their reasonable setting is critical to the result of water treatment. This paper aimed to study the optimal conditions of QSEC; first, single-factor tests were conducted to explore the optimal range of influencing factors, followed by response surface methodology (RSM) tests to accurately determine the optimum values of significant factors. The results show that the addition of quartz sand did not improve the water quality of the coagulation treatment, it took only 140 s for the floc to sink to the bottom, and the sediment volume only accounted for 12.2% of the total sewage. The quartz sand dosage, the coagulant dosage, and sewage pH all had a significant impact on the coagulation effect, and resulted in inflection points. A QSEC-guiding model was derived through RSM tests, and subsequent model optimization and experimental validation revealed the optimal conditions for treating domestic sewage as follows: the polyaluminum chloride (PAC) dosage, cationic polyacrylamide (CPAM) dosage, the sewage pH, quartz sand dosage, stirring time, and settling time were 0.97 g/L, 2.25 mg/L, 7.22, 2 g/L, 5 min, and 30 min, respectively, and the turbidity of the treated sewage was reduced to 1.15 NTU.

## 1. Introduction

Coagulation technology is widely employed in current domestic sewage treatment practices [1,2,3]. However, conventional coagulation has many shortcomings. Firstly, the settling of flocs generated by coagulation is too slow and the settling time is too long, resulting in a large volume and occupied area of the sedimentation tank, which increases construction investment [4,5]. Secondly, research has shown that the efficiency of sewage coagulation treatment (SCT) is significantly related to the dosage of coagulants, that is, increasing the dosage of coagulants within a certain range can effectively improve the efficiency of SCT, but also increase the cost of water treatment [6,7].

In order to improve the above-mentioned shortcomings of conventional SCT, heavy media with fine particles such as magnetic powder and quartz sand are often used to improve its treatment efficiency [8,9,10]. The principle of the improvement is that the heavy media are added to the conventional coagulation process, generating flocs with the heavy media particle as the core, and due to the characteristics of heavy and compact flocs, they can rapidly settle at the bottom of the sedimentation tank. Therefore, this improvement method has the advantages of shortening the coagulation sedimentation time, improving the water treatment capacity of the coagulation sedimentation tank, and reducing its construction volume and cost [11]. Most sewage has the colloidal property of negative charge, and it is often treated by the combined coagulation of polyaluminum chloride and cationic polyacrylamide [12,13,14]. The coagulation effect is influenced by various factors, including the dosage of the coagulant, the dosage and particle size of the heavy medium, settling time, stirring time, sewage pH, etc. Therefore, it is crucial to appropriately adjust these parameters in order to achieve optimal coagulation results [15,16,17]. Only when each factor is set appropriately can the flocculant have the best effect. Consequently, it is necessary to study the factors influencing the coagulation effect and their influencing rule.

Magnetic powder is currently the most commonly used heavy medium because it cannot only reduce sedimentation time but also improve water quality to a certain extent; however, its application is limited due to its high cost [18]. Quartz sand has similar functions to magnetic powder in theory, but its price is significantly cheaper. If quartz sand can be used instead of magnetic powder, it can significantly reduce the processing cost of the coagulation method improved with heavy media. The method of using quartz sand as a heavy medium to enhance the SCT effect is termed the quartz sand-enhanced coagulation (QSEC) method. Moreover, centrifugal separation technology enables the separation and reuse of quartz sand from coagulation flocs, enhancing the application prospects of QSEC.

The response surface methodology (RSM) offers a good test in optimizing and verifying the scientific research [19]. The multivariate quadratic regression equation is utilized to establish the relationship between the optimization objective and influencing factors through regression equation analysis. This methodology aims to identify optimal process parameters and maximize information yield while minimizing experimental efforts [20]. The Box–Behnken design (BBD), a kind of RSM design, is the most frequently used design in pioneering studies due to its scientificity compared with other designs in RSM [21].

At present, there is no report on the improvement of the conventional SCT effect with quartz sand. In order to make up for the shortcomings of the existing research, this study selected polyaluminum chloride (PAC) as the flocculant, cationic polyacrylamide (CPAM) as the coagulant aid, and quartz sand as the heavy medium for the coagulation treatment of domestic sewage. Firstly, a preliminary study, namely a single-factor experiment, was conducted to explore the influencing factors and their optimal range that affect the treatment effect. Then, response surface methodology (RSM) testing was conducted to accurately determine the optimal values of significant factors and obtain the best treatment effect.

## 2. Materials and Equipment

The sewage for coagulation treatment research was collected from the domestic sewage of the student dormitories of Nanyang Normal University. The collected sewage had to be analyzed and treated according to the designed treatment plan within the same day. The water quality of the raw sewage had a turbidity range of 180–352 NTU and a COD range of 450–700 mg/L. In order to maintain the comparability of the test results, the raw sewage was diluted to a chemical oxygen demand (COD) of 200 mg/L before being used for experimental treatment, but different batches of collected sewage had different turbidity values, which ranged from 80 to 150 NTU after dilution.

Cationic polyacrylamide (the aluminum oxide content is approximately 30%) and Cationic polyacrylamide (the molecular weight is approximately 1,200,000) were purchased from Gongyi Yiqing Water-Purifying Material Co., Ltd. (Gongyi, China). Quartz sand (the polyaluminum oxide content is approximately 90%) was purchased from Beijing Yinuo Green Technology Co., Ltd. (Beijing, China), and had three particle sizes, namely 26–40, 70–110, and 200–300 mesh. The hydrochloric acid (HCl) and sodium hydroxide (NaOH) used in this study were of analytical grade. All aqueous and standard solutions were prepared using homemade deionized water.

## 3. Single Factor QSEC Test

### 3.1. Single Factor Test Design

**Figure 1:**
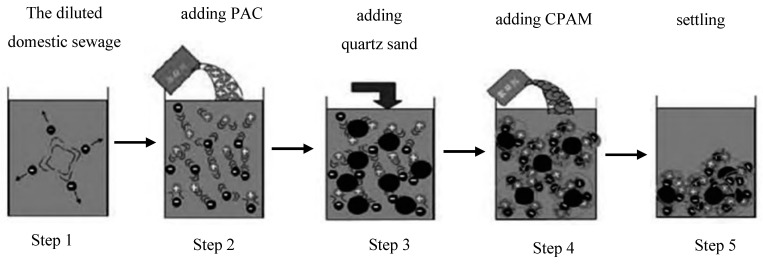
Coagulation treatment process.

In order to investigate the effects of particle size and dosage of quartz sand, coagulation dosage, settling time, and sewage pH on QSEC efficiency, gradient coagulation tests were conducted for each factor to determine their optimal ranges and provide data for subsequent RSM test design. The sewage QSEC treatment process is illustrated in Figure 1, comprising the following five steps:

Step 1: Add 1 L of diluted sewage to a beaker;

Step 2: Add the prepared PAC solution to the sewage and stir with a ZR4-6 coagulation experiment blender (Shenzhen Zhongrunshui Industrial Technology Development Co., Ltd., Shenzhen, China) at a stirring speed of 300 rpm for 2 min to initiate the coagulation reaction;

Step 3: During the stirring period, add quartz sand to the sewage;

Step 4: Add the prepared CPAM solution to the sewage and continue stirring at the stirring speed of 180 rpm for 3 min to induce flocculation reaction;

Step 5: Settle the treated sewage to facilitate the settling of flocs.

During the coagulation treatment process, change the values of influencing factors such as the coagulation dose, the dose and particle size of quartz sand, settling time, and sewage pH during the processing, and study the impact of these factors on the coagulation effect. The turbidity concentration of sewage was measured with the turbidity meter (WZS-186 turbidimeter, Shanghai Yi Electrical Scientific Instrument Co., LTD, China), and the COD was measured with the rapid digested spectrophotometry method, and Uv–vis intelligent multi-parameter water quality tester (Beijing Lianhua Yongxing Technology Development Co., Ltd., Beijing, China) was required in the detection process, and the interface height changes in the sludge layer were observed and recorded.

To investigate the impact of quartz sand on coagulation, the conventional coagulation method was employed as a control. The process did not include the addition of quartz sand, and all other operations were identical to those of the quartz sand-enhanced coagulation (QSEC).

### 3.2. Results and Discussion of Single-Factor Test

#### 3.2.1. Impact of the Particle of Quartz Sand on QSEC

**Figure 2:**
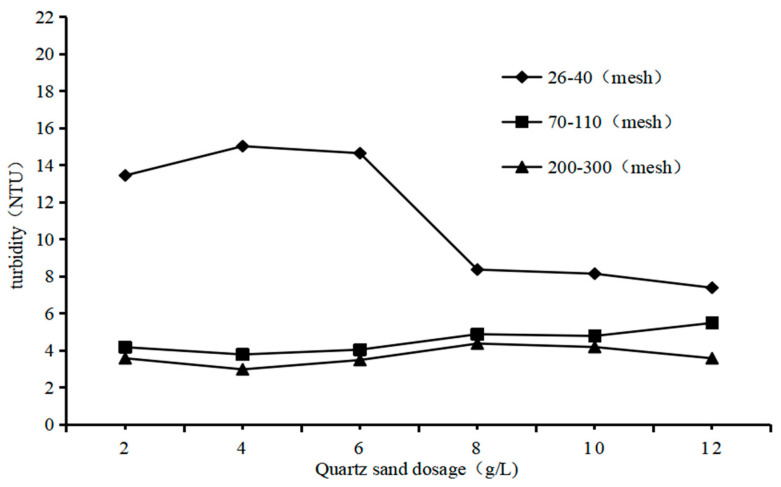
Impact of the particle of quartz sand on QSEC.

In order to investigate the influence of quartz sand particles on QSEC, the following experiments were conducted under specific coagulation treatment conditions: the dosages of PAC and CPAM were of 1.35 g/L and 1.8 mg/L, respectively, the stirring time and settling time were of 5 min and 30 min, respectively, and the sewage pH was of 7. Three types of quartz sand with different particle sizes were added according to the predetermined gradient amounts. The turbidity and COD of the supernatant in each treatment experiment were measured and analyzed, with the results presented in Figure 2.

The impact of quartz sand with different particle sizes on sewage coagulation treatment varies, as depicted in Figure 2. Quartz sand with a size of 200 to 300 mesh corresponds to the lowest sewage turbidity, followed by 70 to 110 mesh, and the worst is 26 to 40 mesh. This result proves that quartz sand is not conducive to sewage coagulation treatment, which may be due to the small particle size of quartz sand which makes it difficult for the formed flocs to settle [22]. Therefore, all subsequent experimental studies utilized quartz sand with a particle size ranging from 200 to 300 mesh as the heavy medium to enhance the efficacy of conventional coagulation.

#### 3.2.2. Impact of Dosage of Quartz Sand on QSEC

**Figure 3:**
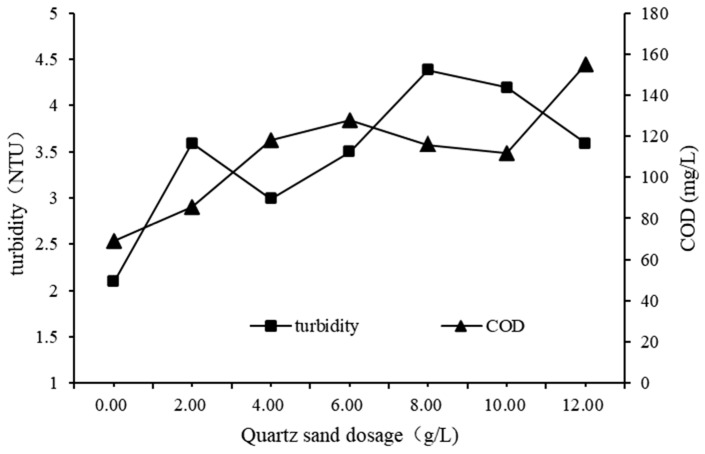
Impact of quartz sand dosage on QSEC.

To investigate the impact of quartz sand dosage on QSEC, a series of experiments were conducted under the following coagulation treatment conditions: PAC and CPAM dosages were set at 1.35 g/L and 1.8 mg/L, respectively, stirring and settling times were maintained at 5 min and 30 min, respectively, sewage pH was adjusted to 7, while varying amounts of quartz sand were added according to predetermined gradient levels. The control group underwent all procedures identical to the experimental group except for the addition of quartz sand. Turbidity and COD levels in the supernatant from each treatment experiment were measured and analyzed, with results presented in Figure 3.

The turbidity and COD levels both demonstrated an upward trend with increasing quartz sand dosage, as depicted in Figure 3. This suggests that excessive use of quartz sand is not conducive to effective coagulation. The reason for this may be that the settling rate of quartz sand is faster than that of flocs, which destroys the structure of flocs and produces some small and difficult to settle flocs, resulting in increased water quality concentration [23]. For cost-saving considerations, the optimal dosage of quartz sand in subsequent experiments was determined to be of 2 g/L.

#### 3.2.3. Impact of PAC Dosage on QSEC

In order to investigate the influence of PAC dosage on QSEC, a series of experiments were conducted under the following coagulation treatment conditions: CPAM and quartz sand dosages were set at 1.8 mg/L and 2 g/L, respectively, with stirring and settling times of 5 min and 30 min, respectively. The pH value of the sewage was maintained at 7, while varying amounts of PAC dosages were added according to predetermined gradient levels. The control group did not include quartz sand, but all other procedures remained identical to those in the experimental group. Turbidity and COD levels in the supernatant from each treatment experiment were measured and analyzed, with results presented in Figure 3.

**Figure 4:**
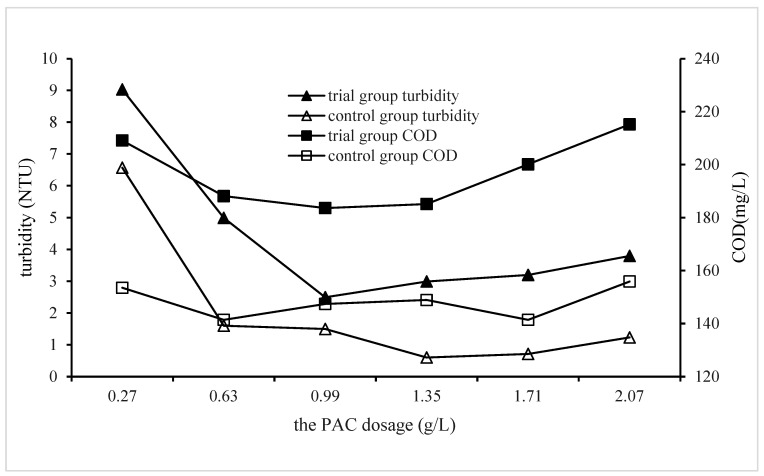
Impact of PAC dosage on QSEC.

The data presented in Figure 4 demonstrate a consistent correlation between the turbidity and COD levels of the experimental group. Specifically, with an increase in PAC dosage, both the turbidity and COD initially decrease to their minimum values before gradually increasing. This phenomenon proved that excessive PAC dosage was not conducive to coagulation. The reason is that excess PAC will react with other ions in the sewage to form substances that are not easy to precipitate, and these substances are re-suspended in the water, causing the water quality to deteriorate. When comparing the coagulation efficiencies of the trial group with those of the control group, it was found that the coagulation efficiency of the control group were visibly better than that of the trial group, and when the PAC dosages were of 1.35 and 1.71 g/L, the sewage turbidity and COD of the control group reduced to the lowest values of 0.6 NTU and 141 mg/L, respectively, while those of the trial group were of 2.5 NTU and 183.6 mg/L, respectively, and the corresponding PAC dosage was of 0.99 g/L. This result once again proves that the addition of quartz sand cannot improve the water quality of coagulation treatment. For cost-saving considerations, the optimal dosage of PAC in subsequent experiments of QSEC was determined to be of 0.99 g/L.

#### 3.2.4. Impact of CPAM Dosage on QSEC

**Figure 5:**
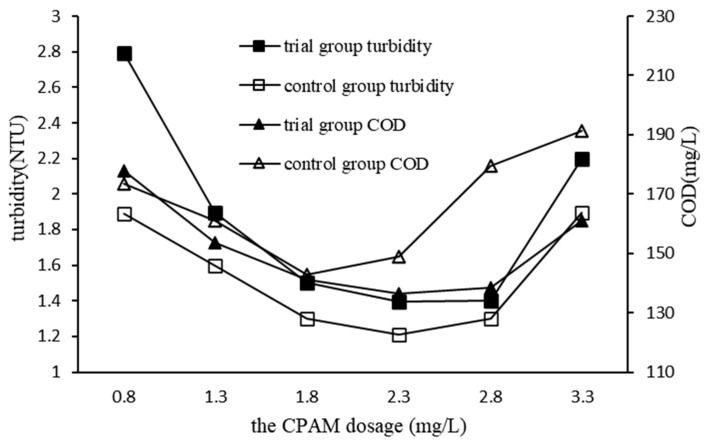
Impact of CPAM dosage on QSEC.

In order to investigate the impact of CPAM dosage on QSEC, a series of experiments were conducted under the following coagulation treatment conditions: PAC and quartz sand dosages were set at 0.99 g/L and 2 g/L, respectively; stirring and settling times were 5 min and 30 min, respectively; sewage pH was maintained at 7. CPAM was added in predetermined gradient amounts. The control group did not add quartz sand, had a PAC dosage of 1.35 g/L, and followed all other procedures identical to the experimental group. The turbidity and COD of the supernatant in each treatment experiment were measured and analyzed, with results presented in Figure 5.

The turbidity and COD of the trial group exhibited a similar trend, as depicted in Figure 5. Specifically, with an increase in CPAM dosage, both the turbidity and COD of the sewage initially decreased to their minimum values before gradually increasing. This phenomenon proved that excessive CPAM dosage was not conducive to coagulation, the reason for which being that excessive CPAM will cause the wastewater to produce positively charged colloidal properties, which will dissolve the precipitated floc, resulting in poor water quality. Comparing the coagulation efficiencies of the trial group with those of the control group, it was found that the coagulation efficiency of the control group was visibly better than that of the trial group, and when the CPAM dosage was of 2.3 mg/L, the turbidity and COD of the trial group reduced to the lowest values of 1.4 NTU and 136.4 mg/L, respectively, while those of control groups were of 1.2 NTU and 142.9 mg/L, respectively, and their corresponding CPAM dosages were of 2.3 and 1.8 mg/L, respectively. For cost-saving considerations, the optimal dosage of CPAM in subsequent experiments of QSEC was determined to be of 2.3 mg/L.

#### 3.2.5. Impact of Sewage pH on QSEC

**Figure 6:**
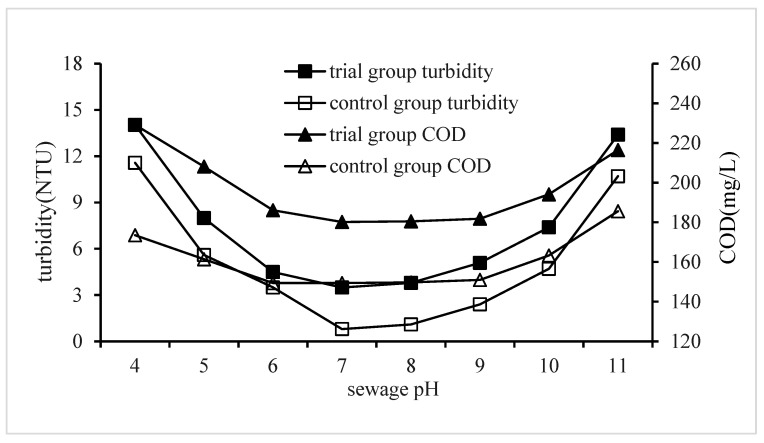
Impact of sewage pH on QSEC.

In order to investigate the influence of sewage pH on QSEC, the following experiments were conducted under coagulation treatment conditions as follows: PAC dosage was of 0.99 g/L, CPAM dosage was of 2.3 mg/L, and quartz sand dosage was of 2 g/L. The stirring time and settling time were set at 5 min and 30 min, respectively. The pH values of the wastewater samples were adjusted according to a predetermined gradient value. For the control groups, no quartz sand was added, while the PAC dosage was increased to 1.35 g/L and the CPAM dosage to 1.8 mg/L; all other procedures remained identical to those in the trial group. Turbidity and COD levels in the supernatant from each treatment experiment were measured and analyzed, with results presented in Figure 6.

Figure 6 illustrates that the changes in COD for both the trial and control groups can be observed as follows: initially, there is a decrease in COD when the pH is below 6; subsequently, it remains stable within a pH range of 6 to 9; finally, an increase in COD occurs when the pH exceeds 9. The minimum COD values for the trial and control groups were of 180.2 and 149.4 mg/L, respectively, with corresponding sewage pH levels of 7 in both cases. The results suggest that sewage pH has a significant impact on coagulation efficiency, with an optimal range of pH between 6 and 9 for effective coagulation. Coagulation performance was found to be poor when the sewage pH fell below 6. The coagulation efficiency was significantly impaired when the pH of the sewage dropped below 6. Several factors contributed to this phenomenon: Firstly, the abundant hydrogen ions in the sewage neutralized the negative charges carried by colloidal particles, resulting in their electrical neutrality or even positive charge, which hindered their coagulation and precipitation [24]. The advantages of charge neutralization offered by CPAM are hindered due to its inability to interact with positive charge groups, thereby exacerbating the electrostatic repulsion between colloidal particles. The decrease in sewage COD primarily stems from the adsorption bridging effect of CPAM [25,26].

The test results also demonstrated that the coagulant performance deteriorated when the pH of the sewage exceeded 9. This phenomenon can be attributed to the abundant hydroxyl ions present in wastewater, which neutralize the positive charge of CPAM and consequently weaken its charge neutralization effect [27,28,29].

The change in turbidity caused by changes in sewage pH is very similar to that of COD, indicating that the mechanism of the pH’s impact on turbidity is the same as that on COD. Under the same treatment conditions, the turbidity of the experimental group was higher than that of the control group. For example, the minimum turbidity values of the experimental group and the control group were of 0.8 and 3.5 NTU, respectively.

#### 3.2.6. Impact of Settling Time on QSEC

**Figure 7:**
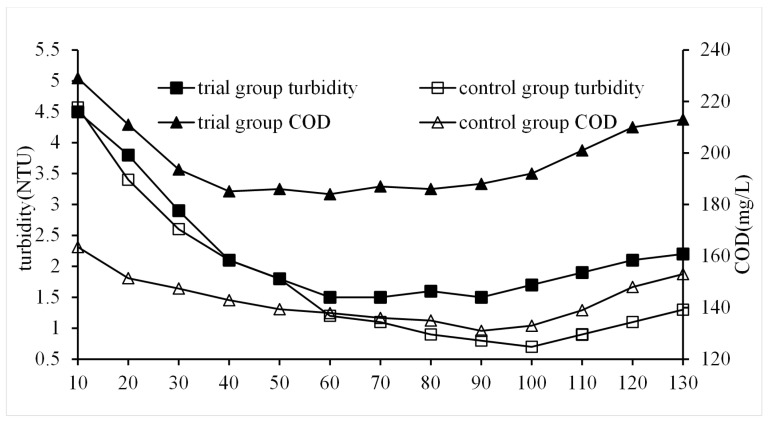
Impact of settling time on QSEC.

In order to study the impact of settling time on QSEC, the following experiments were performed, and the conditions of coagulation treatment were roughly as follows: the dosage of PAC, CPAM, and quartz sand were of 0.99 g/L, 2.3 mg/L, and 2 g/L, respectively, the stirring time was of 5 min, the sewage pH was 7, and the settling times were set according to the predetermined gradient times. The control groups did not add quartz sand, the dosages of PAC and CPAM were of 1.35 g/L and 1.8 mg/L, respectively, and all other procedures were identical to those of the trial group. The turbidity and COD of the supernatant in each treatment experiment were detected and analyzed, and the results are shown in Figure 7.

In order to investigate the influence of settling time on QSEC, a series of experiments were conducted under the following coagulation treatment conditions: PAC dosage of 0.99 g/L, CPAM dosage of 2.3 mg/L, and quartz sand dosage of 2 g/L. The stirring time was set at 5 min, sewage pH was maintained at 7, and settling times were determined based on predetermined gradient intervals. For the control groups, no quartz sand was added and the PAC and CPAM dosages were adjusted to 1.35 g/L and 1.8 mg/L, respectively, while keeping all other procedures identical to those in the trial group. The turbidity and COD in the supernatant from each treatment experiment were measured and analyzed accordingly, and the results are presented in Figure 7.

As can be seen from Figure 7, the COD and turbidity of the trial and control group showed a trend of rapid decline at first, then became stable, and slowly rose at last, indicating that a too long sedimentation time was not conducive to coagulation. The reason for this was that the flocs contain a large amount of organic matter, which fermented and released gas, causing the flocs to float upwards. Throughout the entire precipitation process, the trial group consistently displayed higher COD and turbidity values compared to the control group. After approximately 70 min of settling, the trial group reached its minimum values for COD (184 mg/L) and turbidity (1.5 NTU), whereas it took 90 min for the control group to reach its respective minimum values (133 mg/L for COD and 0.7 NTU for turbidity). This shows that the addition of quartz sand cannot improve the water quality of coagulation treatment, but it can accelerate the coagulation settlement process.

**Figure 8:**
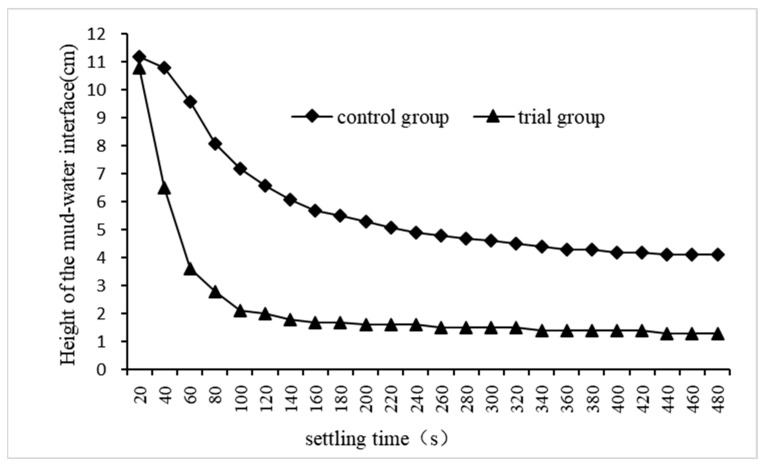
The dynamic changes in the height of the mud–water interface.

We selected the experiments with the best treatment effect in the experimental group and in the control group, recorded their dynamic changes in the height of the mud–water interface, and the results are shown in Figure 8. The height of the mud–water interface first rapidly decreased, and then slowed down until it stopped. The settling rate of floc in the trial group was significantly higher than that in the control group, and the height of the mud–water interface in the former remained stable after 140 s of precipitation, while that in the latter remained stable until 480 s. Throughout the sedimentation process, the mud–water interface height in the trial group consistently remained lower than that in the control group, and at 480 s of sedimentation, floc sedimentation volume accounted for 12.2% of total sewage volume in the trial group, whereas it accounted for 37.4% in the control group. These experimental findings demonstrate that adding quartz sand enhances floc settlement and reduces its volume. This is attributed to the QSEC-formed flocs centered around quartz sand particles with a higher specific gravity, which not only accelerates their settlement but also compresses their volume. Although the addition of quartz sand will lead to the deterioration of the water quality of the coagulation treated sewage, it can accelerate the settlement of flocculates and shorten the time required for coagulation and precipitation. This measure can improve the efficiency of the coagulation sedimentation tank to treat sewage, reduce the construction volume of sedimentation, thus saving the construction cost of the sedimentation tank and reducing the area of the sedimentation tank. Therefore, it will have a good application prospect in engineering practice.

## 4. RSM QSEC Test

### 4.1. RSM QSEC Test Design

A single-factor test generally has the following two shortcomings: one is that the test ignores the influence of the interaction between variable factors on the test results; the other is that the span of the variable value is too large to accurately capture the optimal value of the variable [30,31]. To achieve optimal variable levels and test results, further optimization of the experimental design, such as employing orthogonal design or response surface methodology (RSM), should be conducted based on the outcomes of single-factor tests [32]. The RSM is a highly effective approach for optimizing and validating scientific research as well as industrial studies. It employs multivariate quadratic regression equations to accurately model the relationship between the optimization objective and influencing factors through rigorous regression analysis. This methodology aims to identify optimal process parameters while minimizing the number of experiments required, thereby maximizing information yield. In comparison with orthogonal design, RSM offers enhanced intuitiveness and ease in reflecting the optimal values of dependent variables [33,34]. The Box–Behnken design (BBD), a type of RSM design, is widely employed in pioneering studies due to its superior scientific rigor compared to other RSM designs.

The results of single-factor tests demonstrate that the coagulation process was significantly influenced by the dosage of quartz sand, PAC, CPAM, sewage pH, and settling time. The influences of settling time and quartz sand dosage on the coagulation effect were evident, and their optimal values were determined through a single-factor test. However, the factors, including CPAM dosage, sewage pH, and PAC dosage, exhibited both positive and negative correlations with the coagulation efficiency of CPAM; thus, inflection points were observed where the variable values corresponding to these points were not necessarily optimal but approached optimality. Therefore, based on the results of the single-factor test, a Box–Behnken design (BBD) coagulation test was formulated and executed. The quartz sand with a particle size ranging from 200 to 300 mesh was selected as the heavy medium, and its dosage was of 2 g/L, while the stirring and settling time were of 5 and 30 min, respectively. The sewage identical to that of the single-factor test was treated by coagulation. Reducing the sewage turbidity was the optimization goal, and the CPAM dosage, the sewage pH, and PAC dosage were influencing factors. The BBD test was designed with Design-Expert 13 software, wherein each influence factor was manipulated at three experimental levels: high (+1), low (−1), and central point (basic level 0). The specific values of the independent variables corresponding to these experimental levels were determined and are presented in Table 1. It is noteworthy that all coagulation tests had a uniform settling time of 30 min.

### 4.2. Results and Discussion of the RSM Experiment

#### 4.2.1. Discussion of RSM Experiment Results

According to the BBD test scheme, a total of 17 groups of coagulation tests were conducted, comprising 12 factorial tests and five central tests aimed at inspecting errors. The variable values and the actual turbidity of each coagulation test are presented in Table 2. The results indicate that the turbidities of the five central tests were significantly lower compared to the others, which aligns with the findings from the single-factor test and demonstrates that, while the central point values of the influencing factors in RSM tests were reasonable, they may not necessarily be optimal. Further RSM analysis is required to obtain optimal values [35].

#### 4.2.2. Model Fitting

The quadratic equation model (Y) was formulated based on linear, quadratic, and cross terms as per Equation (1) in the following manner:

Y = A_0_ + A_1_Z_1_ + A_2_Z_2_ + A_3_Z_3_ + A_12_Z_12_ + A_13_Z_13_ + A_23_Z_23_ + A_11_Z_1_^2^ + A_22_Z_2_^2^ + A_33_Z_3_^2^ (1)

Equation (1) represents the correlation between variables and the corresponding response [36]. The term “Y” in this study refers to the response variable that is being modeled, specifically sewage turbidity (NTU); The variables Z_1_, Z_2_, and Z_c_ represent the first-order terms in this study, namely the PAC dosage (g/L), the CPAM dosage (mg/L), and the pH value of sewage. The terms Z_1_^2^, Z_1_^2^, and Z_2_^2^ represent the quadratic components; and the terms Z_12_, Z_13_, and Z_23_ denote the corresponding interaction effects between two variables, respectively, A_0_ was a constant term; A_1_, A_2_, and A_3_ refer to the primary linear coefficients of the PAC dosage (g/L), the CPAM dosage (mg/L) and the sewage pH, respectively; A_11_, A_22_, and A_33_ represent the coefficients of their secondary term, respectively; and A_12_, A_13_, and A_23_ represent the interaction term coefficients among variables, respectively.

The response results of the model were analyzed, and analysis of variance (ANOVA) was utilized to evaluate the feasibility of establishing a quadratic equation model between the variables and responses [37]. The statistical significance of the quadratic equation model and test variables was assessed using F tests and *p* values at a 95% confidence level. The model’s quality was assessed by examining the coefficient of determination R^2^ and adjusted R^2^. Furthermore, the interaction effects of the factors (Z_12_, Z_13_, and Z_23_) on the response variable were analyzed through three-dimensional plots and two-dimensional contour graphs [38].

The regression simulation was performed using the data from Table 2, based on Equation (1), to establish a ternary quadratic polynomial regression model that relates the response to the variables. The resulting equation in terms of actual factors is presented as Equation (2) below.

Y = 118.046 − 14.858Z_1_ − 22.453Z_2_ − 23.37875Z_3_ − 1.806Z_12_ − 0.208Z_13_ + 0.450Z_23_ + 10.513Z_1_^2^ + 4.650Z_2_^2^ + 1.563Z_3_^2^ (2)

The analysis of variance (ANOVA) was performed for the quadratic model of the response surface, as represented by Equation (2). Subsequently, a significance test was conducted to assess the impact of each variable, and the corresponding results are presented in Table 3. The significance of the model terms can be inferred when the “*p* values Prob > F” are less than 0.0500 [39]. In this case, Z_1_, Z_2_, Z_3_, Z_23_, Z_23_, Z_12_, Z_22_, and Z_32_ were all significant model terms, and had significant impacts on sewage turbidity. The model terms Z_1_, Z_2_, Z_3_, Z_23_, Z_23_, Z_12_, Z_22_, and Z_32_ all exhibited significant effects on sewage turbidity in this study. The “*p* values Prob > F” of the model exhibited statistical significance, as their values were below the threshold of 0.0500. The “Lack of Fit F value” of 5.67 implied that the lack of fit was not significant relative to the pure error and indicated that the equation was reliable [40]. The “Pred R-Squared” value of 0.9555 demonstrated reasonable concordance with the “Adj R-Squared” value of 0.9923, indicating that Equation (2) was well-fitted and could be utilized for predicting the turbidity of QSEC. The predicted turbidity values for all QSEC tests based on Equation (2) are presented in Table 2.

The ANOVA analysis revealed that Equation (2) exhibited a satisfactory fitting effect, albeit with a minor limitation: the “*p*-values Prob > F”of Z_13_ exceeded 0.0500, indicating that the interaction between PAC dosage and sewage pH had an insignificant impact on the turbidity of the wastewater. Therefore, the model, namely Equation (2), could be further enhanced by eliminating the intercepts of insignificant terms from the coded model. After optimization, a more refined model was obtained, and its final equation in terms of actual factors is presented as Equation (3).

Y = 119.490 − 16.316Z_1_ − 22.453Z_2_ − 23.585Z_3_ − 1.80556Z_12_ + 0.4503Z_23_ + 10.5133Z_1_^2^ + 4.6503Z_2_^2^ + 1.563Z_3_^2^ (3)

The ANOVA analysis was performed to assess the significance of variable influence in the Response Surface Quadratic Model (Equation (3)). The results presented in Table 3 indicate that the “Lack of Fit F-value” for model, i.e., Equation (3), was approximately 5.38, which is lower than that of Equation (2), suggesting the former’s higher reliability. Therefore, Equation (3) was selected as the predictive model for QSEC turbidity, and the corresponding predicted turbidity values are presented in Table 2.

#### 4.2.3. Response Surface Analysis

**Figure 9:**
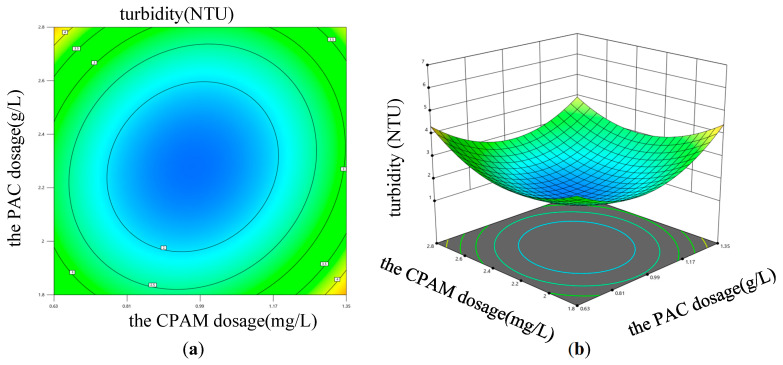
The impact of the interaction between PAC and CPAM dosage on sewage turbidity. (**a**) Contour diagram; (**b**) 3D surface diagram.

**Figure 10:**
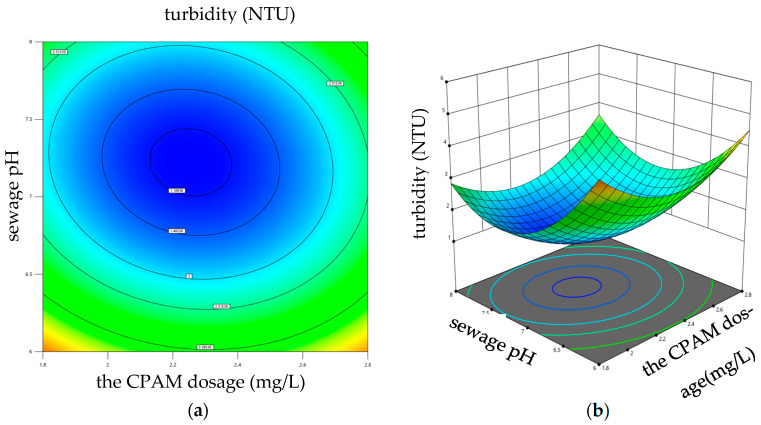
Impact of the interaction between sewage pH and CPAM dosage on sewage turbidity. (**a**) Contour diagram; (**b**) 3D surface diagram.

Equation (3) was employed to conduct ANOVA analysis in order to assess the influence of variable interactions on the coagulation effect. The results reveal that both the interaction between PAC dosage and CPAM dosage, as well as the interaction between sewage pH and CPAM dosage, significantly impacted sewage turbidity. Design Expert 13 software was used to draw the response surface diagrams, which are shown in Figure 9 and Figure 10. The influence of each factor on the sewage turbidity could be judged by the steepness of the three-dimensional response surface. The response surface diagrams, depicted in Figure 9 and Figure 10, were generated using Design Expert 13 software. The steepness of the three-dimensional response surface can be utilized to assess the impact of each factor on sewage turbidity. If the contour graph was oval, it indicated that the interaction effect of the corresponding factors had a significant influence on the sewage turbidity; however, when the contour tended to be circular, the influence was small [41]. The fixed factor was kept constant, while the impacts of the other two factors on the response variable were investigated. The data presented in Figure 9 demonstrate that, as the dosage of PAC and CPAM increases, there is an initial decrease followed by an increase in sewage turbidity under constant pH conditions, and the sewage turbidity reached its minimum value when the dosage of PAC and CPAM ranged from 0.81 to 1.17 g/L and 2 to 2.4 mg/L, respectively. Similarly, as depicted in Figure 10, When the dosage of PAC remained constant, while both the sewage pH and CPAM dosage increased, the turbidity of the sewage exhibited an initial increase followed by a subsequent decrease. The sewage turbidity reached its minimum value when the sewage pH was within the range from 7 to 7.5 and the dosage of CPAM was between 2 and 2.4 mg/L.

#### 4.2.4. Optimal Coagulation Conditions and Model Validation

The first partial derivative of Equation (3) is set to zero to solve, and the optimal solidification conditions are obtained as follows: the dosages of PAC, CPAM, and the sewage pH were of 0.97 g/L, 2.25 mg/L, and 7.22, respectively, and the predicted turbidity value of treated sewage under the above coagulation conditions was 1.11 NTU. To validate the reliability of the prediction model, two additional experimental runs were conducted using the coagulation conditions obtained from model optimization, and other experimental conditions included the selection of quartz sand as the heavy medium, with a particle size ranging from 200 to 300 mesh and a dosage of 2 g/L. The stirring time was set at 5 min, while the settling time was extended to 30 min. The experimental results are presented in Table 4, indicating that the measured turbidities exhibited an average value of 1.12 NTU, which closely corresponds to the predicted value of 1.11 NTU. The discrepancy between the measured turbidity and the predicted turbidity was of merely 3.6%, thereby indicating that the prediction model could serve as a reliable guide for quartz sand-enhanced coagulation. Some scholars use magnetic powder as a heavy medium to improve the effect of traditional coagulation method for water treatment, and its flocculation rate and turbidity are better than the results of this study. However, the price of quartz sand is much lower than that of magnetic powder, making its advantages in reducing the cost of water treatment more obvious [33].

## 5. Conclusions

To investigate the optimal coagulation conditions of QSEC, initial single-factor tests were conducted to preliminarily explore the optimal range of influential factors, followed by subsequent RSM tests to accurately determine the optimum levels of these factors. The results of the single-factor test indicated that the inclusion of quartz sand does not enhance the efficacy of coagulation treatment in improving water quality; however, it does expedite the process of coagulation settlement. The dosage of PAC, CPAM, and the sewage pH all exerted significant influences on the efficacy of QSEC and exhibited optimal levels. A QSEC-guiding model was derived through RSM tests. The results of model optimization reveal the optimal conditions for treating domestic sewage as follows: the PAC dosage, the CPAM dosage, the sewage pH, quartz sand dosage, stirring time, and settling time should be of 0.97 g/L, 2.25 mg/L, 7.22, 2 g/L, 5 min, and 30 min, respectively, and the turbidity of treated sewage is reduced to 1.15 NTU.

## Figures and Tables

**Table 1:** The levels of independent test variables in the experimental study.

Variable Code	Variables	Variable Levels and Corresponding Values		
		−1	0	1
Z_1_	PAC dosage (g/L)	0.63	0.99	1.35
Z_2_	CPAM dosage (mg/L)	1.8	2.3	2.8
Z_3_	sewage pH	6	7	8

**Table 2:** The actual response values and predicted response values of BDD tests.

Run	The CPAM Dosage (mg/L)	The Sewage pH	The Stirring Time (min)	The Response Value of Turbidity (NTU)		
				Actual	Predicted	
					Equation (2)	Equation (3)
1	0.99	2.3	7.0	1.21	1.20	1.20
2	0.63	2.3	6.0	4.60	4.68	4.68
3	0.99	1.8	6.0	4.69	4.68	4.68
4	0.99	2.3	7.0	1.09	1.20	1.20
5	0.63	2.3	8.0	3.31	3.33	3.33
6	1.35	2.8	7.0	3.68	3.68	3.68
7	0.99	2.3	7.0	1.20	1.20	1.20
8	1.35	2.3	8.0	3.49	3.58	3.58
9	1.35	2.3	6.0	5.09	4.93	4.93
10	0.99	2.8	8.0	3.62	3.63	3.63
11	0.63	2.8	7.0	4.19	4.08	4.08
12	0.99	1.8	8.0	3.02	2.88	2.88
13	1.35	1.8	7.0	3.89	4.03	4.03
14	0.63	1.8	7.0	3.11	3.13	3.13
15	0.99	2.8	6.0	4.38	4.53	4.53
16	0.99	2.3	7.0	1.21	1.20	1.20
17	0.99	2.3	7.0	1.31	1.20	1.20

**Table 3:** ANOVAs for the response surface of Equations (2) and (3).

Source		Sum of Squares	Df	Mean Squares	F value	*p*-Value Prob > F	Remark
Model	Equation (2)	31.13	9	3.46	230.56	<0.0001	significant
	Equation (3)	31.1	8	3.89	243.95	<0.0001	significant
Z_1_-the PAC dosage (g/L)	Equation (2)	0.125	1	0.125	8.33	0.0234	
	Equation (3)	0.125	1	0.125	7.84	0.0232	
Z_2_-the CPAM dosage (mg/L)	Equation (2)	0.18	1	0.18	12	0.0105	
	Equation (3)	0.18	1	0.18	11.29	0.0099	
Z_3_-the sewage pH	Equation (2)	3.64	1	3.64	243	<0.0001	
	Equation (3)	3.64	1	3.64	228.71	<0.0001	
Z_12_	Equation (2)	0.4225	1	0.4225	28.17	0.0011	
	Equation (3)	0.4225	1	0.4225	26.51	0.0009	
Z_13_	Equation (2)	0.0225	1	0.0225	1.5	0.2603	
	Equation (3)	-----	-----	-----	-----	-----	
Z_23_	Equation (2)	0.2025	1	0.2025	13.5	0.0079	
	Equation (3)	0.2025	1	0.2025	12.71	0.0074	
Z_1_^2^	Equation (2)	7.82	1	7.82	521.1	<0.0001	
	Equation (3)	7.82	1	7.82	490.44	<0.0001	
Z_2_^2^	Equation (2)	5.69	1	5.69	379.34	<0.0001	
	Equation (3)	5.69	1	5.69	357.03	<0.0001	
Z_3_^2^	Equation (2)	10.28	1	10.28	685.31	<0.0001	
	Equation (3)	10.28	1	10.28	644.99	<0.0001	
Residual	Equation (2)	0.105	7	0.015			
	Equation (3)	0.1275	8	0.0159			
Lack of Fit	Equation (2)	0.085	3	0.0283	5.67	0.0635	not significant
	Equation (3)	0.1075	4	0.0269	5.38	0.0661	not significant
Pure Error	Equation (2)	0.02	4	0.005			
	Equation (3)	0.02	4	0.005			
Cor Total	Equation (2)	31.23	16				
	Equation (3)	31.23	16				
R^2^_Pre_	Equation (2)	0.9555					
	Equation (3)	0.9603					
R^2^_adj_	Equation (2)	0.9923					
	Equation (3)	0.9918					

**Table 4:** Measured and predicted values of sewage turbidity.

Coagulation Conditions						Sewage Turbidity (NTU)	
PAC Dosage (g/L)	CPAM Dosage (mg/L)	Sewage pH	Quartz Sand Dosage (g/L)	Stirring Time (min)	Settling Time (min)	Average of Measured Value	Predicted Value
0.97	2.25	7.22	2	5	30	1.15	1.11

## Data Availability

The data supporting this article have been included as part of the article.
